# Ethical Challenges in Delivering Surgical Innovation: Laparoscopic Bariatric-Metabolic Surgery and Sentinel Node Biopsy for Melanoma: an Australian Perspective

**DOI:** 10.1007/s11695-023-06504-7

**Published:** 2023-02-27

**Authors:** John B Dixon, Stuart J Anderson, Jeffery Hamdorf

**Affiliations:** 1 Iverson Health Innovation Research Institute, Swinburne University of Technology, Melbourne, Australia; 2 General Practitioner, Maffra Medical Group, Maffra, Australia; 3 Professor of Surgical Education & Director CTEC, The University of Western Australia, Perth, Australia

**Keywords:** Ethics, Surgery, Innovation, Review, Outcomes

The Australian public must have confidence that innovative surgical interventions are safe, effective, and justifiable economically. Surgical innovation involves risks. Informed consent is challenging when the risk to benefit ratio is unknown, and especially so when the objectivity of the surgeon-innovators, colleagues, universities, and hospitals is compromised by conflict of interest. The primary areas of conflict for those involved in surgical innovation are economic, but prestige, career advancement, optimism bias, and reputation for being at the cutting edge are all at play [1]. By its nature, innovation in surgery usually precedes a full evaluation of key health outcomes. Two examples of surgical innovation from the 1990s are contrasted.

Society’s narratives regarding the management of cancer and obesity are strikingly different. Cancer is discussed positively reflecting hope and optimism. There is positive engagement from the health sector, and advocacy groups and forums provide patient support and advice. Cancer therapy is discussed alongside health outcomes and survival. In contrast, obesity is considered a burden, a problem reflecting fear and pessimism. Obesity management focuses on the individual, usually through behavior change. Advocacy, public support, and access to effective therapies are found wanting. Weight stigma is pervasive and effective therapies are often perceived unnecessary, ineffective, and unsafe [2–4].

Bariatric-metabolic surgery (BMS) remains the most effective therapy for Australians with clinically severe obesity and its related complications including type 2 diabetes, cardiovascular disease, and many cancers.

The evidence supporting efficacy, safety, improved quality of life and survival, and cost-effectiveness of established laparoscopic surgical procedures is clear [5, 6]. However, fewer than 2% of Australians with an indication for surgery access treatment annually. Ninety-four percent of this surgery takes place in private hospitals [7]. The opportunity for systemic life-changing, lifesaving surgery is evidently not a public health service priority. This lack of access to surgical care occurs in an environment where none of the growing range of safe and effective weight management pharmaceuticals is listed within Pharmaceutical Benefits Scheme. Timely, and equitable, access to care for Australians with clinically severe obesity, and its numerous biopsychosocial complications, is not considered and the voices of those with lived experience are rarely heard. The science and biology of the ongoing pandemic of obesity tells us this narrative must change. Shaming and blaming have not and, will not work, and cause harm [3].

In contrast, surgical innovation in cancer management is embraced with enthusiasm with assumptions of safety, efficacy, and cost-effectiveness. The procedure of sentinel lymph node biopsy (SLNB) and, when positive, completion lymph node dissection and removal, provides a salient example. SLNB was developed to detect early regional lymph node melanoma involvement and hence prolong survival. It became a standard of practice, widely included in guidelines. The multicenter selective lymphadenectomy trial (MSLT-1) tested the hypothesis that SLNB would save lives [8]. The 10-year results showed no difference in melanoma-related mortality. A second study MSLT-2 tested the hypothesis that regional lymph node clearance following a positive sentinel node would reduce melanoma mortality. Again, there was no mortality reduction in those having the more extensive procedure [9]. These results suggested the sentinel node was not a gate keeper and that a surgical approach beyond the appropriate primary wide excision of the cutaneous melanoma was not effective in altering the natural history of what had become a systemic disease.

However, a positive SLNB improves the prediction of melanoma mortality marginally (C-statistic only 3%) beyond that of established clinical and pathologic criteria [10]. Currently any role for SLNB is unclear, but the complications of nodal biopsy and clearance are established and may cause permanent impairment. Protagonists strongly defend SLNB, arguing that it may provide prognostic information to assist in selecting patients for targeted systemic therapy, and they still believe SLNB is a standard of care. As a result, this costly procedure is provided regularly across Australia despite clear evidence of risk without evidence of benefit [10]. Indications for targeted therapy to manage patients at higher risk of melanoma mortality should be focused established clinical and pathologic criteria and, on the efficacy, and safety, and specificity of individual therapies.

More than one million Australian adults live with clinically severe obesity. Unless we can change the narrative and embrace the biology and science, most will die without receiving any effective medical or surgical interventions for their serious progressive chronic disease.

The uptake and equitable delivery of surgical innovation should be based on evidence that interventions improve the lives of Australians, and not be subject to bias, value judgments, or presumption. An ongoing program of health economic evaluation is needed to verify the role of innovation in surgery.

## Data Availability

All data is available in the accompanying references.

## References

[1] Johnson J, Rogers W. Innovative surgery: the ethical challenges. J Med Ethics. 2012;38:9-12. jme.2010.042150 .10.1136/jme.2010.042150 10.1136/jme.2010.042150 21697295

[2] Flint SW (2020). The NHS long-term plan: a comparison of the narrative used for cancer and obesity. Lancet Diabetes Endocrinol.

[3] Rubino F, Puhl RM, Cummings DE, Eckel RH, Ryan DH, Mechanick JI, Nadglowski J, Ramos Salas X, Schauer PR, Twenefour D (2020). Joint international consensus statement for ending stigma of obesity. Nat Med.

[4] Scudellari M (2015). The science myths that will not die. Nature..

[5] Lee PC, Dixon J (2017). Bariatric-metabolic surgery: A guide for the primary care physician. Aust Fam Physician.

[6] Syn NL, Cummings DE, Wang LZ, Lin DJ, Zhao JJ, Loh M, Koh ZJ, Chew CA, Loo YE, Tai BC (2021). Association of metabolic-bariatric surgery with long-term survival in adults with and without diabetes: a one-stage meta-analysis of matched cohort and prospective controlled studies with 174 772 participants. Lancet.

[7] Bariatric Surgery Registry 8th annual report. Monash University. https://www.monash.edu/__data/assets/pdf_file/0004/2582131/2021-Bariatric-Surgery-Registry_8th-Annual-Report_Amended_May.pdf. 2021. Accessed 22 June.

[8] Morton DL, Thompson JF, Cochran AJ, Mozzillo N, Nieweg OE, Roses DF, Hoekstra HJ, Karakousis CP, Puleo CA, Coventry BJ (2014). Final trial report of sentinel-node biopsy versus nodal observation in melanoma. New Eng J Med.

[9] Faries MB, Thompson JF, Cochran AJ, Andtbacka RH, Mozzillo N, Zager JS, Jahkola T, Bowles TL, Testori A, Beitsch PD (2017). Completion Dissection or Observation for Sentinel-Node Metastasis in Melanoma. New Eng J Med.

[10] El Sharouni MA, Stodell MD, Ahmed T, Suijkerbuijk KPM, Cust AE, Witkamp AJ, Sigurdsson V, van Diest PJ, Scolyer RA, Thompson JF (2021). Sentinel node biopsy in patients with melanoma improves the accuracy of staging when added to clinicopathological features of the primary tumor. Ann Oncol.

